# *Toxoplasma* IWS1 Determines Fitness in Interferon-γ-Activated Host Cells and Mice by Indirectly Regulating ROP18 mRNA Expression

**DOI:** 10.1128/mbio.03256-22

**Published:** 2023-01-30

**Authors:** Emi Hashizaki, Miwa Sasai, Daisuke Okuzaki, Tsubasa Nishi, Takashi Kobayashi, Shiroh Iwanaga, Masahiro Yamamoto

**Affiliations:** a Department of Immunoparasitology, Research Institute for Microbial Diseases, Osaka University, Suita, Osaka, Japan; b Department of Molecular Parasitology, Research Institute for Microbial Diseases, Osaka University, Suita, Osaka, Japan; c Laboratory of Immunoparasitology, WPI Immunology Frontier Research Center, Osaka University, Suita, Osaka, Japan; d Department of Immunoparasitology, Center for Infectious Disease Education and Research, Osaka University, Suita, Osaka, Japan; e Genome Information Research Center, Osaka University, Suita, Osaka, Japan; f Department of Medical Zoology, Faculty of Medicine, Mie University, Mie, Japan; g Infectious Disease Control, Faculty of Medicine, Research Center for Global and Local Infectious Diseases, Oita University, Oita, Japan; Stanford University

**Keywords:** IWS1, ROP18, IFN-γ, parasitophorous vacuole, virulence, *Toxoplasma*, interferons, transcriptional regulation, virulence factors

## Abstract

Toxoplasma gondii secretes various virulence effector molecules into host cells to disrupt host interferon-γ (IFN-γ)-dependent immunity. Among these effectors, ROP18 directly phosphorylates and inactivates IFN-inducible GTPases, such as immunity-related GTPases (IRGs) and guanylate-binding proteins (GBPs), leading to the subversion of IFN-inducible GTPase-induced cell-autonomous immunity. The modes of action of ROP18 have been studied extensively; however, little is known about the molecular mechanisms by which ROP18 is produced in the parasite itself. Here, we report the role of T. gondii transcription factor IWS1 in ROP18 mRNA expression in the parasite. Compared with wild-type virulent type I T. gondii, IWS1-deficient parasites showed dramatically increased loading of IRGs and GBPs onto the parasitophorous vacuole membrane (PVM). Moreover, IWS1-deficient parasites displayed decreased virulence in wild-type mice but retained normal virulence in mice lacking the IFN-γ receptor. Furthermore, IWS1-deficient parasites showed severely decreased ROP18 mRNA expression; however, tagged IWS1 did not directly bind with genomic regions of the ROP18 locus. Ectopic expression of ROP18 in IWS1-deficient parasites restored the decreased loading of effectors onto the PVM and *in vivo* virulence in wild-type mice. Taken together, these data demonstrate that T. gondii IWS1 indirectly regulates ROP18 mRNA expression to determine fitness in IFN-γ-activated host cells and mice.

## INTRODUCTION

Toxoplasma gondii is an obligatory protozoan parasite that can infect cells with nuclei from virtually all warm-blooded animals (1). Although it is estimated that one-third of the world’s population is infected with T. gondii, most infections are asymptomatic. Infection with T. gondii, however, can lead to life-threatening toxoplasmosis in immunocompromised humans and animals (2). Moreover, T. gondii is ranked among the top five human pathogens which cause economic loss and life impairment via foodborne illness in the United States (3). Thus, T. gondii is an important human and animal pathogen.

T. gondii forms parasitophorous vacuoles (PVs) in infected cells, which are membranous structures originating from host cell plasma membranes and are generated during invasion by the parasite. T. gondii can proliferate only inside the PV membrane (PVM), which is an interface between the host and the parasite (1). During infection, T. gondii secretes various molecules from secretory organelles such as rhoptry and dense granules into the host cell cytoplasm and nucleus, onto the PVM, and within the PV space (4). One important function of rhoptry proteins (ROPs) and dense granule proteins (GRAs) is downregulating the host immunity that is dependent on interferon gamma (IFN-γ) (5). IFN-γ robustly stimulates the expression of hundreds of genes encoding a variety of proteins related to anti-T. gondii cell-autonomous immunity (4, 6). IFN-γ-stimulated nitric oxide production and tryptophan degradation are important for suppression of T. gondii growth (7, 8). IFN-inducible GTPases such as p47 immunity-related GTPases (IRGs) and p65 guanylate-binding proteins (GBPs) are required for disruption of the PVM of avirulent type II T. gondii to kill the pathogen in a cell-autonomous fashion (9–11). IRGs and GBPs are coordinately accumulated on the T. gondii PVM. Among these, Irgb6 has been shown to act as a pioneer which detects T. gondii PVM, leading to the recruitment of other IRGs such as Irga6 and Irgb10, GBPs, and effectors, including p62/Sqstm1 and ubiquitin (12, 13). Irgb6-deficient mice are susceptible to type II T. gondii to the same extent as IFN-γ-deficient mice (13). Thus, Irgb6 plays a fundamental role in IFN-γ-dependent anti-T. gondii cell-autonomous host defense.

Targeting Irgb6 is one of the most effective virulence mechanisms of virulent type I T. gondii (14, 15). Rhoptry kinases, such as ROP17 and ROP18, and a pseudokinase, ROP5, are secreted into the host cell cytoplasm and localized at the PVM to directly phosphorylate and inactivate IFN-inducible GTPases, inhibiting their accumulation on the T. gondii PVM (16, 17). Loss of ROP18 from a virulent type I T. gondii strain results in decreased virulence in mice (17, 18). Thus, targeting host Irgb6 via ROP18 is an important counterdefense system for virulent T. gondii in IFN-γ-activated cells. However, little is known about the mechanism by which ROP18 is prepared at the transcriptional, translational, and post-translational levels inside type I T. gondii itself.

A genome-wide loss-of-function screen in a type I T. gondii strain identified >350 genes which determine fitness in IFN-γ-activated murine macrophages (19). Some of the highly ranked genes, such as GRA45, TGGT1_263560 and TGGT1_269950 (encoding putative GRAs), were characterized to determine the fitness-related mechanisms (19). However, most of the remaining genes are uncharacterized so far. Here, to explore the mechanisms by which T. gondii-derived molecules target host Irgb6-dependent cell-autonomous immunity, we tested the involvement of several type I T. gondii proteins encoded by uncharacterized genes in the suppression of Irgb6-dependent host defense. We found that a putative transcription factor, IWS1, is important for ROP18-mediated virulence of type I T. gondii. Loss of IWS1 from type I parasites led to markedly increased accumulation of Irgb6 and other IFN-inducible GTPases at the PVM, and severely decreased the parasite virulence in mice. Global gene profile analysis revealed that IWS1 deficiency resulted in the downregulation of various genes, including the ROP18 gene. Moreover, ROP18 overexpression in IWS1-deficient T. gondii reduced recruitment of IFN-inducible GTPases to the PVM and restored *in vivo* virulence in wild-type mice. In conclusion, this study demonstrates that the T. gondii transcription factor IWS1 is important for ROP18 mRNA expression in the virulent parasite to subvert host IFN-inducible GTPase-mediated cell-autonomous immunity; this determines the fitness of the parasite in IFN-γ-activated cells and mice.

## RESULTS

### Increased Irgb6 loading on the PVM of IWS1-deficient type I *T. gondii*.

**FIG 1:**
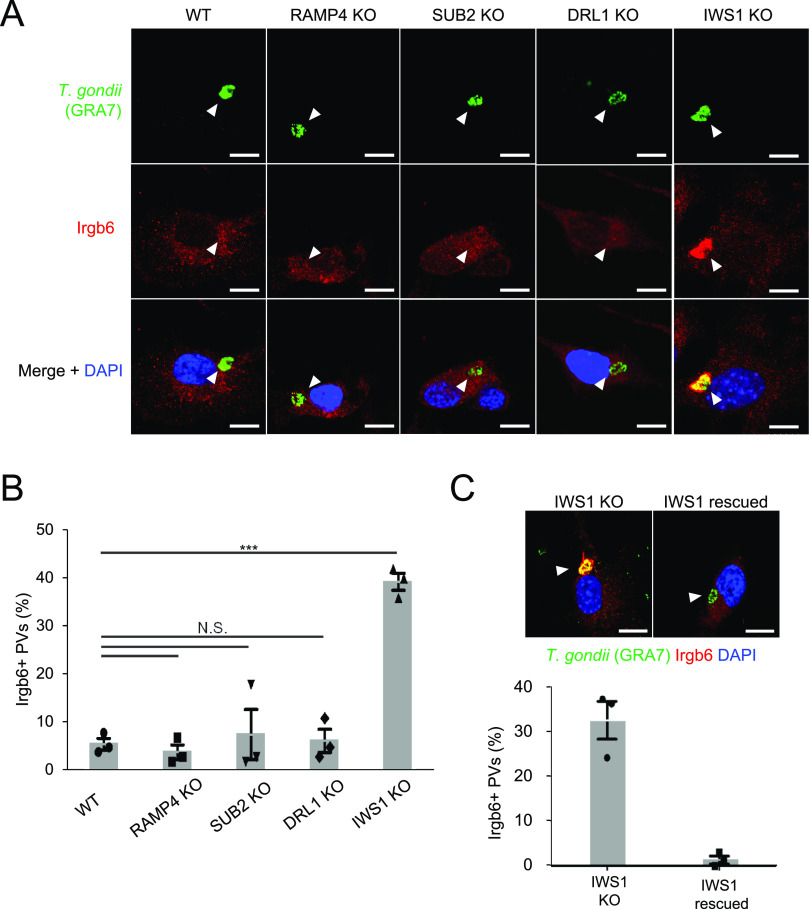
IWS1 deletion increased loading of Irgb6 onto Toxoplasma gondii parasitophorous vacuole membrane (PVM). (A and B) Confocal microscope images (A) and graphs (B) represent the localization of Irgb6 (red) to T. gondii vacuoles (green) and DAPI (4′,6-diamidino-2-phenylindole; blue) at 2 h postinfection of wild-type (WT) or indicated knockout (KO) parasites in mouse embryonic fibroblasts (MEFs) treated with 10 ng/mL IFN-γ. A total of 100 PVs were counted in each experiment. White arrowheads indicate parasitophorous vacuoles (PVs). Scale bars = 10 μm. (C) Confocal microscope images (upper) and graphs (lower) represent the localization of Irgb6 (red) to T. gondii vacuoles (green) and DAPI (blue) at 2 h postinfection with IWS1 KO or IWS1-rescued parasites in MEFs treated with 10 ng/mL IFN-γ. A total of 100 PVs were counted in each experiment. Scale bars = 10 μm. All images are representative of three independent experiments (A and C). Indicated values are means ± standard deviation (SD) of three biological replicates (B and C). ***, *P* < 0.001; N.S., nonsignificant.

Except for *gra45*, *gra22*, *TGGT1_263560*, and *TGGT1_269950*, the candidate genes which determine T. gondii fitness in IFN-γ-stimulated murine macrophages have not been tested for Irgb6 recruitment (19). The proteins encoded by *gra45*, *gra22*, *TGGT1_263560*, and *TGGT1_269950* are GRAs. GRA family proteins have been extensively studied (20). Thus, to find new mechanisms of T. gondii virulence, we chose non-GRA genes such as *TGGT1_269620* (encoding a DNA repair-like protein, DRL1), *TGGT1_314500* (encoding a protease, SUB2), *TGGT1_276940* (encoding a putative ribosome-associated membrane protein, RAMP4), and *TGGT1_227560* (encoding a putative transcription factor, IWS1) from among the candidates (19). We explored possible roles of these non-GRA proteins in subversion of host Irgb6-mediated, anti-T. gondii cell-autonomous immunity. We generated type I parasites lacking DRL1, SUB2, RAMP4, and IWS1, respectively, by CRISPR/Cas9 genome editing (Fig. S1A and B). Consistent with previous CRISPR screening reports (19, 21), type I T. gondii lacking DRL1, SUB2, RAMP4, or IWS1 formed plaques in a manner similar to wild-type parasites (Fig. S1C). Then, we tested the mutant parasites for Irgb6 recruitment to the PVM in IFN-γ-stimulated mouse embryonic fibroblasts (MEFs) (Fig. 1A and B). The percentages of Irgb6-coated PVM were comparable to those for wild-type parasites for the DRL1-, SUB2-, and RAMP4-deficient parasites (Fig. 1A and B). Notably, however, IWS1-deficient T. gondii showed much higher levels of Irgb6 recruitment to the PVM than the wild-type or the other gene-disrupted parasites (Fig. 1A and B). To assess whether the increased Irgb6 accumulation on the PVM of IWS1-deficient parasites was due to the lack of IWS1, we complemented IWS1 into the endogenous locus in IWS1-deficient parasites (Fig. 4D and Fig. S2A) and tested Irgb6 loading onto the PVM (Fig. 1C). The percentage of Irgb6 recruitment to the PVM of IWS1-reconstituted IWS1-deficient parasites was significantly less than that for the IWS1-deficient parasites (Fig. 1C), indicating that IWS1, but not DRL1, SUB2, or RAMP4, is involved in subverting the Irgb6-dependent anti-T. gondii response of host cells.

10.1128/mbio.03256-22.1 FIG S1 Generation of Toxoplasma gondii lacking RAMP4, SUB2, DRL1 or IWS1. (A) Deletion of genes. Wild-type locus of each gene was cleaved by CRISPR/Cas9 at the indicated gRNA target sites, and a PCR fragment containing the *hxgprt* cDNA with two flanking *loxP* sequences was inserted. (B) Quantitative reverse transcription-PCR (RT-PCR) analysis of parasites. Gene expression levels of RAMP4, Drl1, SUB2, and IWS1 in wild-type (WT) and knockout (KO) parasites were normalized relative to TgACT1 expression. Data are representative of two independent experiments (C) Microscope images of plaques in crystal violet-stained monolayer of mouse embryonic fibroblasts (MEFs). Black arrowheads indicate plaques. All images are representative of three independent experiments. Related to Fig. 1. Download FIG S1, PDF file, 0.5 MB. Copyright © 2023 Hashizaki et al. 2023 Hashizaki et al. https://creativecommons.org/licenses/by/4.0/ This content is distributed under the terms of the Creative Commons Attribution 4.0 International license.

**FIG 2:**
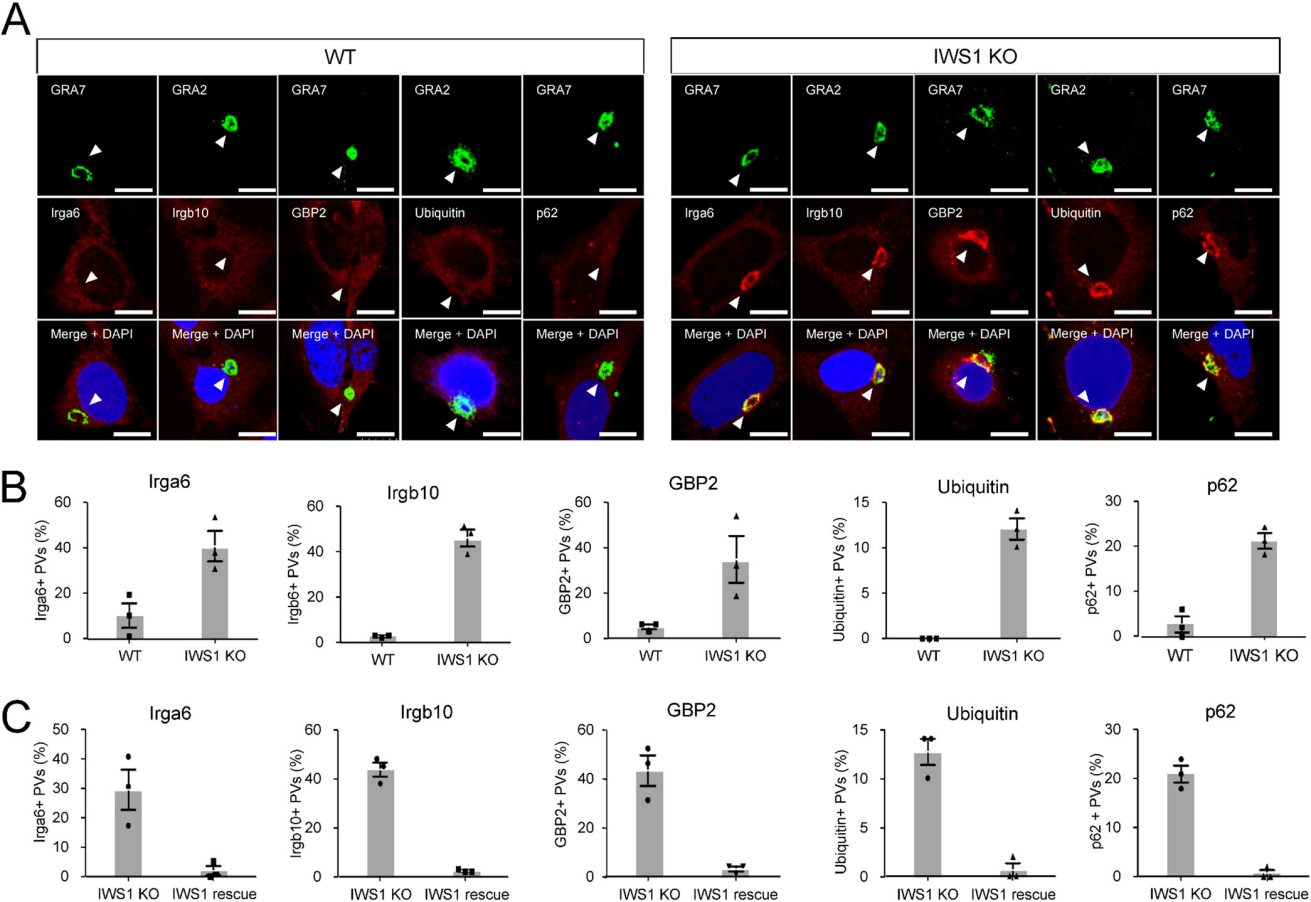
IWS1 deletion increased loading of Irga6, GBP2, ubiquitin, and p62 onto T. gondii PVM. (A and B) Confocal microscope images (A) and graphs (B) represent the localization of Irga6, Irgb10, Gbp2, ubiquitin, and p62 (red) to T. gondii vacuoles (green) and DAPI (blue) at 2 h postinfection with WT or IWS1 KO parasites in MEFs treated with 10 ng/mL IFN-γ. A total of 100 PVs were counted in each experiment. White arrowheads indicate PVs. Scale bars, 10 μm. (C) Recruitment rates of the indicated host proteins on PVs of IWS1 KO or IWS1-rescued parasites at 2 h postinfection with IWS1 KO or IWS1-rescued parasites in MEFs treated with 10 ng/mL IFN-γ. A total of 100 PVs were counted in each experiment. All images are representative of three independent experiments (A). Indicated values are means ± SD of three biological replicates (B and C).

10.1128/mbio.03256-22.2 FIG S2 Strategy of IWS1 complementation in IWS1 KO T. gondii. (A) Complementation of IWS1 KO parasites. The *hxgprt* cassette in the endogenous IWS1 locus was deleted with Cre transfection in the presence of 6-thioxanthine. A PCR fragment containing an IWS1 cDNA copy with a poly A signal and the *hxgprt* cassette was inserted following Cas9 cleavage of the indicated region (arrowhead). (B) ROP18 gene expression cassette driven by the 1 kb of SAG gene promoter. Related to Fig. 2. Download FIG S2, PDF file, 0.06 MB. Copyright © 2023 Hashizaki et al. 2023 Hashizaki et al. https://creativecommons.org/licenses/by/4.0/ This content is distributed under the terms of the Creative Commons Attribution 4.0 International license.

### Increased effector recruitment to the PVM of IWS1-deficient parasites.

Irgb6 recognizes the T. gondii PVM and subsequently elicits downstream recruitment of other effectors, such as Irga6, Irgb10, GBPs, and p62/Sqstm1 (12, 13). Therefore, we next assessed the recruitment of effectors other than Irgb6 to the PVM of wild-type and IWS1-deficient parasites (Fig. 2A and B and Fig. S3). The percentages of loading of IRGs, such as Irga6 and Irgb10, onto the PVM were higher for IWS1-deficient parasites than for wild-type parasites in IFN-γ-stimulated MEFs (Fig. 2A and B and Fig. S3). In addition to IRGs, GBP2, ubiquitin, and p62/Sqstm1 proteins were more frequently detected on the PVM of IWS1-deficent parasites than on the PVM of wild-type parasites (Fig. 2A and B and Fig. S3). Moreover, complementation of IWS1 in IWS1-deficient parasites markedly decreased the recruitment of Irga6, Irgb10, and p62/Sqstm1 to the PVM (Fig. 2C). Collectively, these results demonstrate that T. gondii IWS1 negatively regulates host IFN-γ-inducible GTPase-dependent antiparasitic responses.

10.1128/mbio.03256-22.3 FIG S3 Immunity-related GTPase (IRG) localization on parasitophorous vacuole membrane (PVM) of IWS1-KO parasites. Low magnification confocal microscope images represent the localization of Irgb6, Irga6, Irgb10, and p62 (red) to T. gondii vacuoles (green), and DAPI (blue) at 2 h postinfection with WT or IWS1 KO parasites in MEFs treated with 10 ng/mL IFN-γ. White arrowheads indicate parasitophorous vaculoes (PVs) without IRGs. Arrows indicate PVs with IRGs. Scale bars = 10 μm. All images are representative of three independent experiments. Related to Fig. 2. Download FIG S3, PDF file, 0.3 MB. Copyright © 2023 Hashizaki et al. 2023 Hashizaki et al. https://creativecommons.org/licenses/by/4.0/ This content is distributed under the terms of the Creative Commons Attribution 4.0 International license.

### Decreased *in vivo* virulence of IWS1-deficient type I *T. gondii*.

**FIG 3:**
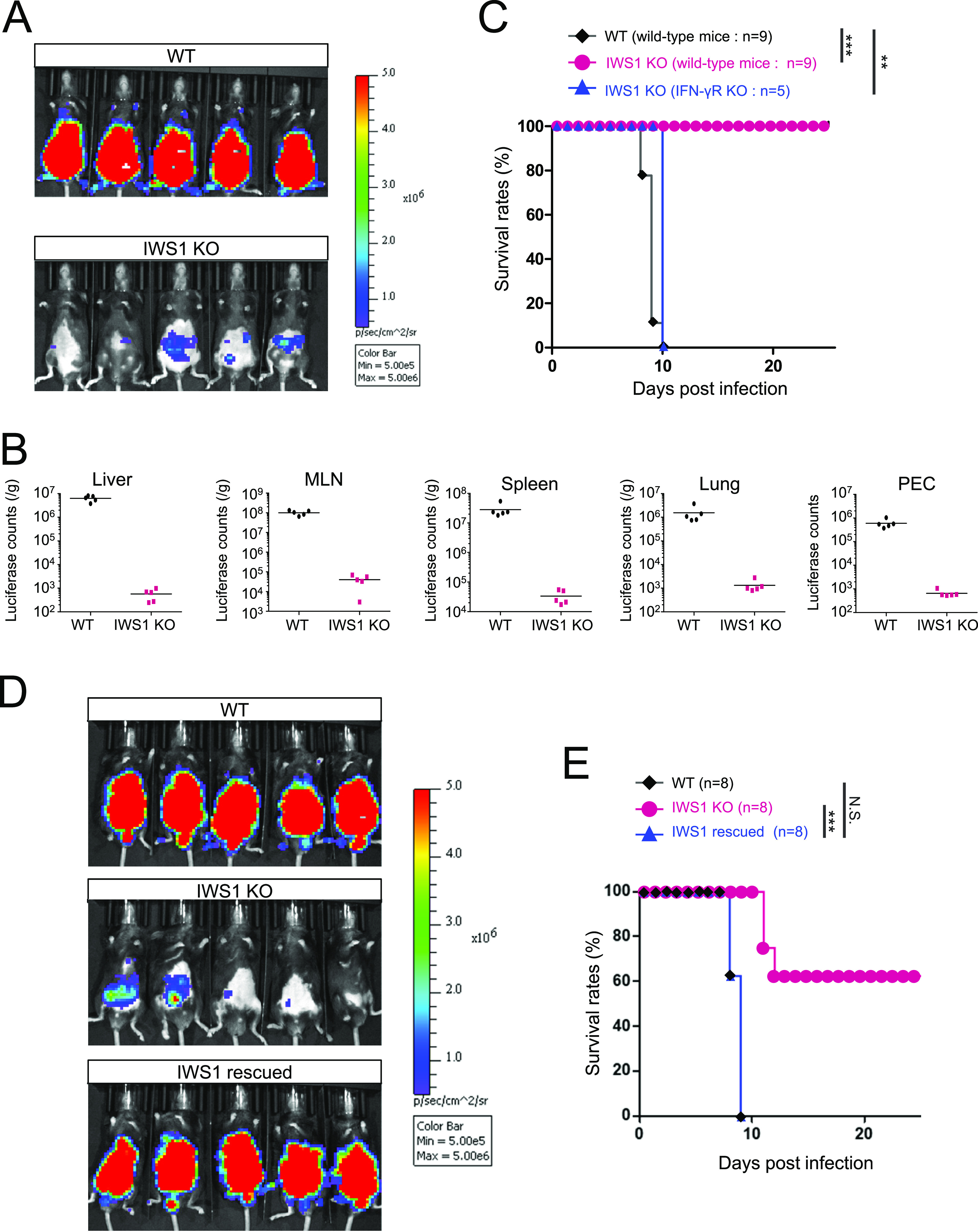
Loss of IWS1 reduced virulence *in vivo*. (A) IVIS imaging of mice infected with WT or IWS1 KO parasites. Images were taken on day 6 postinfection. (B) Luciferase counts in indicated organs of WT parasite-infected (*n* = 5) and IWS1 KO parasite-infected mice (*n* = 5). (C) Survival curves of WT parasite-infected wild-type mice (*n* = 9), IWS1 KO parasite-infected wild-type mice (*n* = 9), and IWS1 KO parasite-infected IFN-γR-deficient mice (*n* = 5). (D) Bioluminescence imaging of mice infected with WT, IWS1 KO, or IWS1-rescued parasites. Images were taken on day 6 postinfection. (E) Survival curves of WT parasite-infected (*n* = 8), IWS1 KO parasite-infected (*n* = 8), and IWS1-rescued IWS1 KO parasite-infected mice (*n* = 8). All images are representative of three independent experiments (A and D). Data are cumulative of two or three independent experiments (B, C and E). ^**^P < 0.01; ***, *P* < 0.001; N.S. nonsignificant.

IWS1 deficiency in type I T. gondii led to defective suppression of IFN-inducible GTPase-mediated cell-autonomous immunity in MEFs, prompting us to examine the effect of IWS1 deficiency on *in vivo* virulence (Fig. 3). Wild-type mice were intraperitoneally infected with luciferase-expressing wild-type or IWS1-deficient T. gondii and examined for parasite spreading using an *in vivo* imaging system (Fig. 3A). Strong abdominal luciferase signals were detected in the mice infected with wild-type parasites; however, the luciferase signals in mice infected with IWS1-deficient parasites were remarkably decreased (Fig. 3A). We next assessed parasite burdens in tissues from mice infected with wild-type or IWS1-deficient parasites (Fig. 3B). At 6 days postinfection, wild-type parasite-injected mice contained much higher parasite burdens in peritoneal cavities and tissues such as the liver, mesenteric lymph nodes, spleen, and lungs than IWS1-deficient parasite-injected mice (Fig. 3B). We next compared the survival of wild-type parasite-infected and IWS1-deficient parasite-infected mice (Fig. 3C). All of the wild-type parasite-infected mice died within 10 days. Mice infected with IWS1-deficient parasites survived for longer than those infected with wild-type parasites (Fig. 3C). Furthermore, IWS1 complementation in IWS1-deficient parasites restored parasite spread and *in vivo* virulence (Fig. 3D and E). Moreover, although wild-type mice showed prolonged survival following IWS1-deficient parasite infection, IFN-γ receptor (IFN-γR)-deficient mice infected with IWS1-deficient parasites succumbed in a similar time period to wild-type parasite-infected wild-type-mice (Fig. 3C). Taken together, these results indicate that T. gondii IWS1 is required for suppression of IFN-γ-dependent host defense *in vivo*.

### Severely decreased ROP18 mRNA expression in IWS1-deficient type I T. gondii.

**FIG 4:**
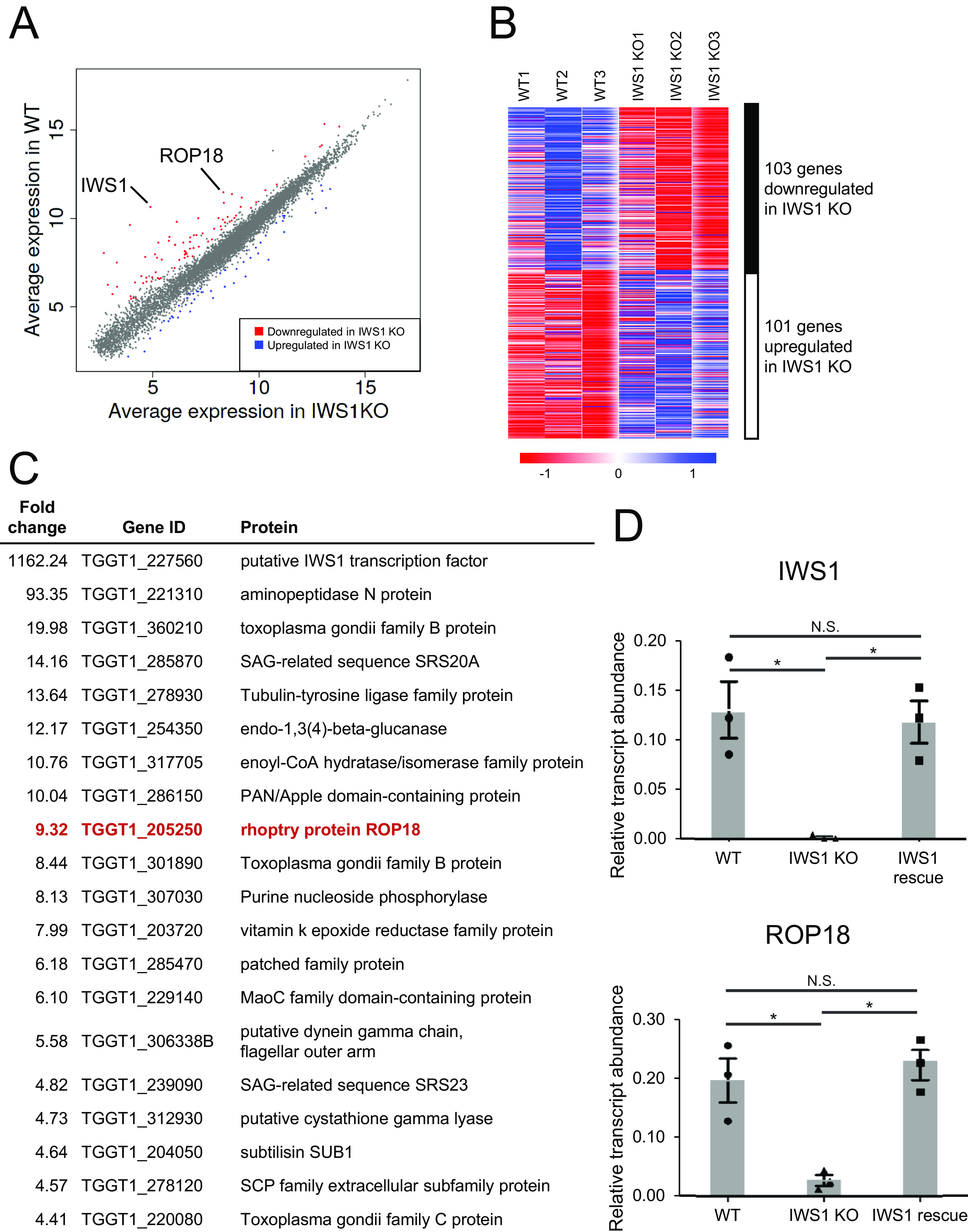
Lower ROP18 mRNA expression in IWS1 KO parasites than in WT parasites. (A to C) RNA-seq transcriptome analysis of WT and IWS1 KO parasites. Analysis was performed with RNA from WT (*n* = 3) and IWS1 KO parasites (*n* = 3). (A) Scatterplot comparing gene expression profiles in WT and IWS1 KO parasites. (B) Heat map illustration of hierarchical clustering of overall gene expression profiles of samples from WT and IWS1 KO parasites. Genes with an average fold change of >2 are shown. (C) List of the top genes (hypothetical proteins excluded) downregulated in IWS1 KO parasites compared with WT parasites, with an average fold change of >4.5. (D) Quantitative reverse transcription-PCR (RT-PCR) analysis of IWS1 and ROP18 expression in WT, IWS1 KO, and IWS1 rescued IWS1 KO parasites. Gene expression was normalized relative to TgACT1 expression. Indicated values are means ± SD of three biological replicates (D). *, *P* < 0.05; N.S., nonsignificant.

We next explored the molecular mechanism(s) by which IWS1 suppresses host IFN-inducible GTPase-mediated cell-autonomous immunity. IWS1 was shown to be a transcription elongation factor in yeast (22). Therefore, we compared global gene expression profiles in freshly isolated tachyzoites of wild-type and IWS1-deficient type I T. gondii (Fig. 4A to C). RNA-seq analysis demonstrated that hundreds of genes were differentially expressed between wild-type and IWS1-deficient parasites (Fig. 4A), with significant upregulation of 101 genes and downregulation of 103 genes in IWS1-deficient parasites relative to the wild-type (*P* < 0.05 and |log_2_-fold change| > 1) (Fig. 4B). IWS1 mRNA was the most differentially expressed gene between wild-type T. gondii and IWS1-deficient parasites (Fig. 4C, Fig. S4, and Table S1). The defective IWS1 mRNA expression was confirmed by quantitative reverse transcription-PCR (RT-PCR; Fig. 4D, upper). Notably, mRNA encoding the virulence factor ROP18, which was shown to inactivate IFN-inducible GTPases (16, 17), was one of the genes highly differently expressed between wild-type T. gondii and IWS1-deficient parasites (Fig. 4C, Fig. S4, and Table S1). When we examined ROP18 mRNA expression by quantitative RT-PCR, IWS1-deficient parasites showed dramatically lower levels of ROP18 mRNA expression than wild-type (WT) parasites (Fig. 4D, bottom). Furthermore, complementation of IWS1 restored mRNA expression of ROP18 and that of IWS1 (Fig. 4D), suggesting that IWS1 is required for ROP18 mRNA expression.

10.1128/mbio.03256-22.4 FIG S4 List of IWS1-dependent genes including hypothetical proteins. List of the top genes (hypothetical proteins included) downregulated in IWS1 KO samples compared with WT samples, with average fold change of >4.5. Related to Fig. 4. Download FIG S4, PDF file, 0.09 MB. Copyright © 2023 Hashizaki et al. 2023 Hashizaki et al. https://creativecommons.org/licenses/by/4.0/ This content is distributed under the terms of the Creative Commons Attribution 4.0 International license.

10.1128/mbio.03256-22.6 TABLE S1 List of IWS1-regulated genes revealed by RNA-seq analysis. Normalized fragments per kilobase of exon per million mapped fragments (FPKM) counts for indicated genes are shown. Fold changes between wild-type and IWS1-deficient parasites are shown in column B with the significance scores in column C. Download Table S1, XLSX file, 2.2 MB. Copyright © 2023 Hashizaki et al. 2023 Hashizaki et al. https://creativecommons.org/licenses/by/4.0/ This content is distributed under the terms of the Creative Commons Attribution 4.0 International license.

### IWS1 indirectly influences ROP18 mRNA expression.

To assess whether IWS1 controls ROP18 mRNA expression directly or indirectly, we conducted genome-wide chromatin immunoprecipitation coupled with high-throughput sequencing (ChIP-seq) based upon the binding site information for IWS1 in the T. gondii genome. First, we generated T. gondii expressing the N-terminally Venus-tagged IWS1 (Fig. S5A). The Venus-IWS1 was detected solely in the parasitic nucleus but not in the cytosol, whereas the control Venus alone was observed in both regions (Fig. S5B). Next, we performed ChIP-seq by anti-GFP (green fluorescent protein) antibody. The obtained DNA was sequenced with the NovaSeq 6000 sequencer, and reads were mapped onto the T. gondii GT-I genome sequence. Three independent ChIP-seq analyses identified peaks in the same regions on the genome (Fig. S5C), suggesting high reproducibility in the replicates. When we examined the ROP18 locus in the mapped view, no peaks were observed on the promoter, coding, and 5′- or 3′-untranslated regions of the ROP18 gene (Fig. S5D), indicating that the Venus-IWS1 does not directly bind with the ROP18 gene regions. We found 453 common peaks on the T. gondii GT-I genome in all three replicates (Table S2A). Furthermore, putative IWS1 direct target genes with the common peaks included nine genes encoding known AP2 family transcription factors (Table S2B). Among them, TgAP2VIII-7 mRNA expression level was 23% lower in IWS1-deficient parasites compared with wild-type parasites (Tables S1 and S2). Taken together, the ChIP-seq analyses indicated that IWS1 indirectly influences ROP18 mRNA expression.

10.1128/mbio.03256-22.5 FIG S5 Nuclear localization of Venus-IWS1 and ChIP-seq (chromatin immunoprecipitation coupled with high-throughput sequencing) analysis. (A) Insertion of the HXGPRT/SAG1p-Venus cassette into the N terminus of IWS1 by genome editing. (B) Confocal microscope images represent the localization of Venus (green) to T. gondii (red) and DAPI (blue) at 2 h postinfection with Venus-IWS1 parasites or control parasites expressing Venus alone. Scale bars = 10 μm. (C) Comparison of the mapped views of Venus-IWS1 peaks obtained in three independent experiments (experiments 1, 2, and 3). The views were created with an integrative genomics viewer. Reads were mapped on the reference sequence of a region of T. gondii GT-I chromosome Ia. (D) Venus-IWS1 peaks were not observed in the ROP18 gene locus. The image was created by the integrative genomics viewer. All images are representative of three independent experiments (B, C and D). Related to Fig. 4. Download FIG S5, PDF file, 0.3 MB. Copyright © 2023 Hashizaki et al. 2023 Hashizaki et al. https://creativecommons.org/licenses/by/4.0/ This content is distributed under the terms of the Creative Commons Attribution 4.0 International license.

10.1128/mbio.03256-22.7 TABLE S2 Analysis of direct IWS1-binding sites and IWS1 target genes. (A) Peaks identified in the ChIP-seq (chromatin immunoprecipitation coupled with high-throughput sequencing) analysis of IWS1 in experiment 1. Whether the peaks were detected in experiments 2 and 3 is shown in columns K and L, respectively. (B) List of putative target genes of IWS1. Gene ID and product names are shown. Download Table S2, XLSX file, 0.2 MB. Copyright © 2023 Hashizaki et al. 2023 Hashizaki et al. https://creativecommons.org/licenses/by/4.0/ This content is distributed under the terms of the Creative Commons Attribution 4.0 International license.

### Ectopic ROP18 expression overcomes IWS1 deficiency.

**FIG 5:**
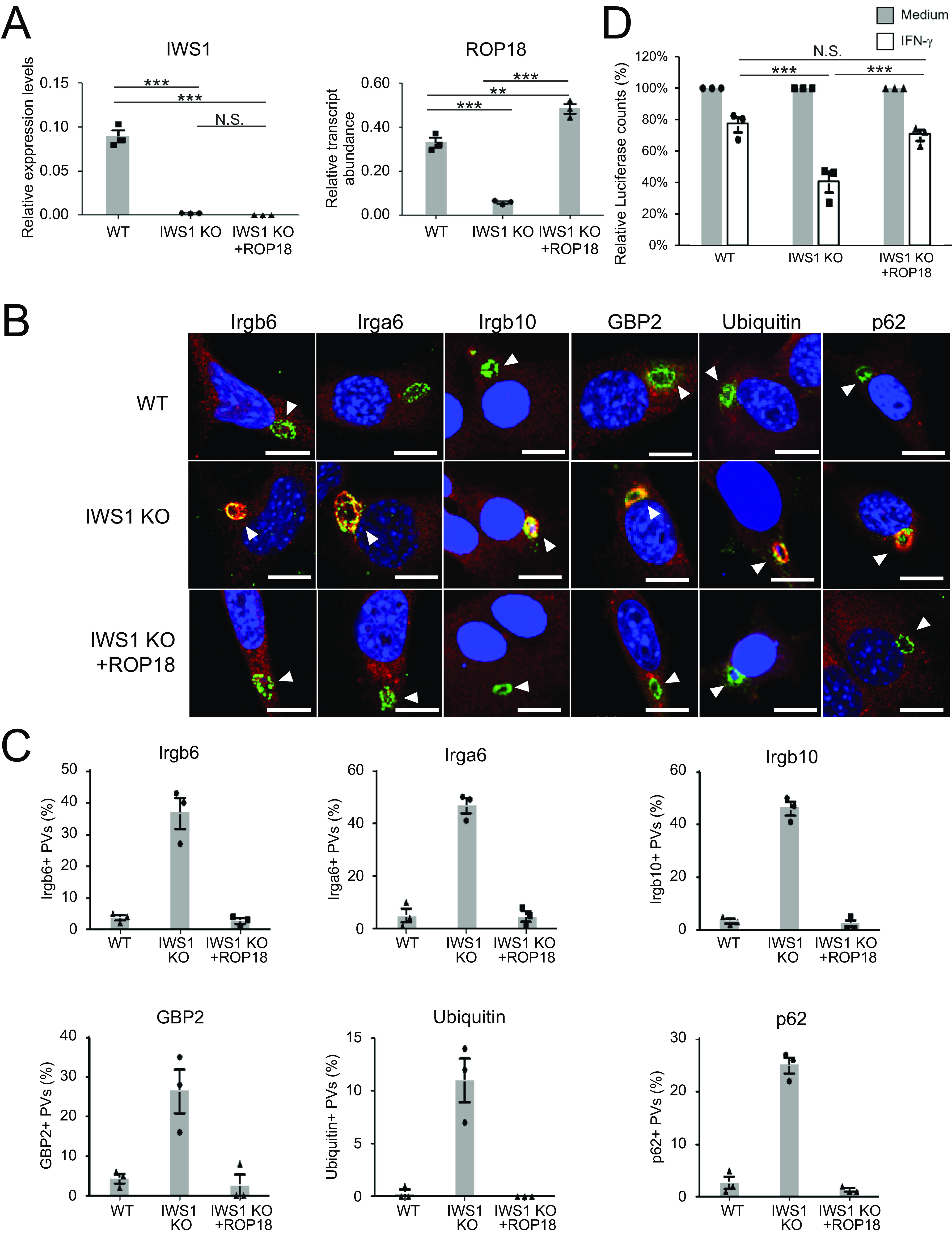
Overexpression of ROP18 in IWS1 KO parasites reduced effector recruitment onto the PVM. (A) Quantitative RT-PCR analysis of WT, IWS1 KO, or ROP18-overexpressing IWS1 KO parasites (IWS1KO+ROP18). Gene expression was normalized relative to TgACT1 expression. (B and C) Confocal microscope images (B) and graphs (C) represent the localization of Irga6, Irgb10, Gbp2, ubiquitin, and p62 (red) to T. gondii vacuoles (green) and DAPI (blue) at 2 h postinfection with WT, IWS1 KO, or ROP18-overexpressing IWS1 KO parasites (IWS1KO+ROP18) in MEFs treated with 10 ng/mL IFN-γ. A total of 100 PVs were counted in each experiment. White arrowheads indicate PVs. Scale bars = 10 μm. (D) Relative luciferase counts of IFN-γ-treated or untreated cells infected with WT, IWS1 KO, or ROP18-overexpressing parasites (IWS1KO+ROP18). Data are the percentage of luciferase counts in IFN-γ-treated/untreated samples of indicated parasite lines. All images are representative of three independent experiments (B). Indicated values are means ± SD of three biological replicates (A, C and D). **, *P* < 0.01; ***, *P* < 0.001; N.S., nonsignificant.

It remained unclear whether the severely decreased ROP18 mRNA expression when IWS1 was absent led to the defective suppression of host IFN-inducible GTPase-mediated cell-autonomous immunity by the parasite. To clarify the causal relationship, we examined whether IWS1-independent ROP18 overexpression could overcome the effect of IWS1 deficiency. A plasmid vector which allowed ectopic expression of ROP18 under the SAG1 promoter was transfected into IWS1-deficient parasites. We confirmed higher ROP18 mRNA levels in ROP18-overexpressing IWS1-deficient parasites than in IWS1-deficient parasites, but the ROP18-overexpressing IWS1-deficient parasites still lost IWS1 mRNA expression (Fig. 5A). Then, we compared recruitment of IFN-inducible GTPases, ubiquitin, and p62/Sqstm1 to the PVM of IWS1-deficient parasites, ROP18-overexpressing IWS1-deficient parasites, and wild-type parasites (Fig. 5B and C). The PVM of IWS1-deficient parasites was highly coated with Irgb6, Irga6, Irgb10, Gbp2, ubiquitin, and p62/Sqstm1. However, coating with IFN-inducible GTPases and effectors was profoundly decreased on the PVM of ROP18-overexpressing IWS1-deficient parasites and wild-type parasites (Fig. 5B and C). The number of IWS1-deficient parasites in IFN-γ-stimulated MEFs was lower than the number of wild-type parasites. On the other hand, infection of MEFs with IWS1-deficient parasites overexpressing ROP18 led to impaired IFN-γ-induced reduction of parasite cell numbers, which was reminiscent of infection with wild-type parasites (Fig. 5D). Taken together, these results demonstrate that ectopic ROP18 expression restored suppression of IFN-inducible GTPase-mediated cell-autonomous responses to IWS1-deficient parasites *in vitro*.

### ROP18 overexpression in IWS1-deficient parasites considerably restores *in vivo* virulence.

**FIG 6:**
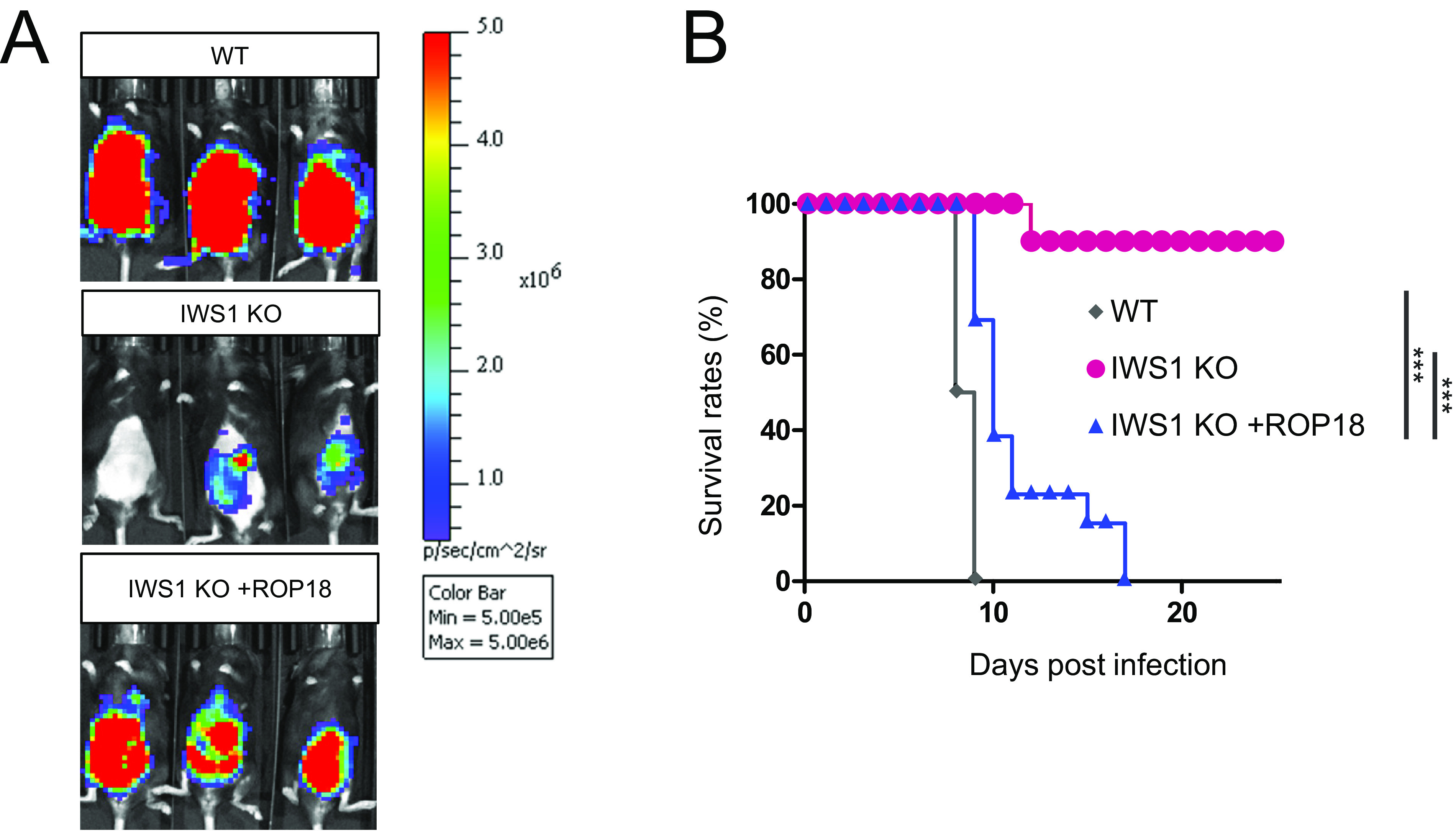
Overexpression of ROP18 in IWS1 KO parasites considerably recovered virulence *in vivo*. (A) Bioluminescence imaging of mice infected with WT, IWS1 KO, or ROP18-overexpressing (IWS1KO+ROP18) parasites. Images were taken on day 6 postinfection. (B) Survival curves of WT parasite-infected (*n* = 10), IWS1 KO parasite-infected (*n* = 10), or ROP18-overexpressed IWS1 KO (IWS1KO+ROP18) parasite-infected mice (*n* = 13). All images are representative of three independent experiments (A). Data are cumulative of two or three independent experiments (B). ***, *P* < 0.001.

Finally, we assessed whether the IWS1-independent ROP18 overexpression could also restore *in vivo* virulence. To assess parasite spread, wild-type mice were intraperitoneally infected with luciferase-expressing wild-type, IWS1-deficient, or ROP18-overexpressing IWS1-deficient parasites. We found that the luciferase signals from mice infected with ROP18-overexpressing IWS1-deficient parasites were greater than those mice infected with IWS1-deficient parasites, but less than those from mice infected with wild-type parasites (Fig. 6A). Next, we assessed the survival of infected mice (Fig. 6B). Consistent with the *in vivo* parasitic spread (Fig. 6A), mice infected with ROP18-overexpressing IWS1-deficient parasites survived for significantly longer than those infected with wild-type parasites, but not as long as those infected with IWS1-deficient parasites (Fig. 6B). Collectively, these data show that IWS1-independent ROP18 overexpression considerably restored the *in vivo* virulence of IWS1-deficient T. gondii.

## DISCUSSION

In the present study, we searched for T. gondii factors which can inhibit the Irgb6-dependent cell-autonomous response in genes that confer fitness in IFN-γ-stimulated murine cells (19). In a previous study, two putative GRA proteins (TGGT1_263560 and TGGT1_269950) were shown to be involved in suppressing Irgb6 loading on the T. gondii PVM by unknown mechanisms. We considered that the candidate GRA proteins, except for TGGT1_263560 and TGGT1_269950, and even non-GRA proteins might be inhibitors of Irgb6-mediated host defense. Among the non-GRA proteins, we have identified a putative IWS1 transcription elongation factor as a new inhibitor of Irgb6-dependent anti-T. gondii cell-autonomous immunity. Moreover, the T. gondii IWS1 was important for ROP18 mRNA expression. IWS1 was originally discovered as an RNA polymerase II elongation factor in yeast (22). IWS1 is shown to interact with the conserved elongation factor Spt6 (also known as SUPT6) to regulate H3K36 trimethylation and play crucial roles in mRNA processing and export (23). Currently, it remains unclear whether the T. gondii IWS1 also functions as a transcription elongation factor. The ChIP-seq analysis suggested that the T. gondii IWS1 might indirectly regulate transcription of ROP18 mRNA. The parasite IWS1 may indirectly induce ROP18 mRNA by expressing other transcription factors that directly transcribe ROP18 mRNA. Given the results of RNA-seq and ChIP-seq analyses, TgAP2VIII-7 might be involved in initiating transcriptional cascades leading to ROP18 mRNA expression downstream of IWS1. Alternatively, we cannot exclude the possibility that indirect binding of IWS1 to the promoter of *rop18* through the association with other transcription factors such as the parasite Spt6, RNA polymerase II and TgAP2XI-5 which can directly bind to the *rop18* promoter (24).

We hypothesized that environmental stress could change IWS1 gene expression levels and hence affect ROP18 expression levels. However, when we monitored IWS1 expression levels in various culture conditions such as IFN-γ-stimulation, low pH, and oxidative stress, the IWS1 levels were unchanged compared to those in unstimulated conditions (data not shown), suggesting that type I parasites may not regulate ROP18 mRNA levels via IWS1 in response to the conditions tested. When IWS1 amino acid sequences were compared in type I (TGGT1_227560), type II (TGME49_227560) and type III (TGVEG_227560), only one amino acid was different, suggesting that the polymorphic IWS1 function might be unlikely. Although we could not find changes in IWS1 gene expression in cultured tachyzoites, transcriptomic databases published in previous studies showed that stable IWS1 mRNA expression was detected in the bradyzoite and oocyst stages of the parasite life cycle (25–27). On the other hand, ROP18 mRNA is not expressed in the oocyst stage (25–27), which is inconsistent with the IWS1 expression pattern. The discrepant mRNA expression profile between IWS1 and ROP18 in the oocyst stage suggests that not only IWS1 but also other stage-specific transcription factors may be required for ROP18 mRNA expression in the tachyzoite and bradyzoite stages. Alternatively, oocyst-specific transcriptional repressors may downregulate IWS1-dependent ROP18 mRNA expression in the oocyst stage.

ROP18-overexpressing IWS1-deficient parasites appeared to fully reconstitute suppression of IFN-inducible GTPase recruitment to the parasite *in vitro* and considerably rescued *in vivo* virulence in mice. However, infection with ROP18-overexpressing IWS1-deficient parasites still resulted in a minor prolonged survival period in mice, indicating the contribution of IWS1-dependent ROP18-independent gene product(s) to the remaining *in vivo* virulence. Given that IFN-γR-deficient mice were susceptible to IWS1-deficient parasites in a similar time course to wild-type mice infected with wild-type T. gondii, IWS1-dependent gene product(s) other than ROP18 may be involved in subversion of IFN-γ-dependent host defense. Among the mRNAs encoding various named proteins and uncharacterized hypothetical proteins that were reduced by 1.5-fold in IWS1-deficient parasites, genes encoding the SAG-related sequence SRS29B (TGGT1_233460) and two hypothetical proteins (TGGT1_280375 and TGGT1_289740) were also reported to significantly confer fitness in IFN-γ-stimulated murine macrophages, albeit without high confidence (19). Although the roles of SAG-related sequence SRS29B (TGGT1_233460) and two hypothetical proteins (TGGT1_280375 and TGGT1_289740) in T. gondii virulence remain uncertain, these proteins might suppress IFN-γ-dependent but IFN-inducible GTPase-independent host defense for full virulence *in vivo*.

In conclusion, we have identified a parasite protein which regulates the transcription of ROP18 mRNA, by which type I T. gondii can subvert IFN-inducible GTPase-dependent cell-autonomous immunity to determine fitness in IFN-γ-activated cells. ROP18 has IWS1 for transcriptional production machinery. Similarly, other ROP and GRA virulence effectors may have their own or shared production and trafficking machineries within this pathogen, such as TgASP5, which is a parasite Golgi-resident protease that cleaves several GRAs to localize the PVM and host nucleus (28). Considering as conventional virulence factors not only secreted and injected effectors but also the machineries responsible for their specific expression might enable us to change our interpretation of fitness genes identified in CRISPR screens (19, 29), provide new insights into T. gondii immune-parasitological knowledge, and possibly give us new therapeutic targets for treatment and prevention of toxoplasmosis.

## MATERIALS AND METHODS

### Parasites and mice.

C57BL/6NCrSlc (C57BL/6N) mice were purchased from SLC. Mice lacking IFN-γR have been previously described (30). All animal experiments were conducted with the approval of the Animal Research Committee of Research Institute for Microbial Diseases at Osaka University. RHΔ*hxgprt*Δ*ku80* and its derivatives of T. gondii were maintained in Vero cells by bi-weekly passage in RPMI (Nacalai Tesque) supplemented with 2% heat-inactivated fetal calf serum (FCS; JRH Bioscience), 100 U/mL penicillin and 0.1 mg/mL streptomycin (Nacalai Tesque). Mouse embryonic fibroblasts were maintained in Dulbecco’s modified Eagle medium (DMEM; Nacalai Tesque) supplemented with 10% heat-inactivated FCS, 100 U/mL penicillin, and 0.1 mg/mL streptomycin (Nacalai Tesque).

### Reagents.

Goat polyclonal antibody against Irgb6 (TGTP; sc-11079) was purchased from Santa Cruz Biotechnology, Inc. Rabbit polyclonal anti-GBP2 and mouse monoclonal anti-p62 (H00008878-M01J) antibodies were obtained from Proteintech and Abnova, respectively. An anti-ubiquitin rabbit monoclonal antibody (Apu2; Merck) was obtained from Nippon Biotest Laboratories. A mouse monoclonal anti-Irga6 (10D7) antibody was provided by J.C. Howard (Instituto Gulbenkian de Ciencia). Mouse monoclonal anti-GRA2 antibody and rabbit polyclonal anti-GAP45 antibody were provided by D. Soldati-Favre (University of Geneva). A rabbit polyclonal anti-GRA7 antibody was provided by John C. Boothroyd (Stanford University). Recombinant mouse IFN-γ was purchased from PeproTech.

### Plasmid construction for generation of knockout *T. gondii* strains.

For construction of the CRISPR/Cas9 plasmids targeting IWS1 (TGGT1_227560), SUB2 (TGGT1_314500), RAMP4 (TGGT1_276940) or DRL1 (TGGT1_269620), two oligonucleotide primers (IWS1_gRNA1_F and IWS1_gRNA1_R, IWS1_gRNA2_F and IWS1_gRNA2_R for IWS1, SUB2_gRNA1_F and SUB2_gRNA1_R, SUB2_gRNA2_F and SUB2_gRNA2_R for SUB2, RAMP4_gRNA1_F and RAMP4_gRNA1_R, RAMP4_gRNA2_F and RAMP4_gRNA2_R for RAMP4, DRL1_gRNA1_F and DRL1_gRNA1_R, DRL1_gRNA2_F and DRL1_gRNA2_R for DRL1) containing gRNA sequence were annealed and cloned into the BsaI site of the pU6-Universal vector. The primer sequences are listed in Table S3. To generate a construct for deleting the entire coding sequence of IWS1, SUB2, RAMP4 or DRL1, 60-bp flanking regions of 5′ outside and 3′ outside the gRNAs were used to surround the genes. The targeting fragments containing floxed *hxgprt* cassette used primers IWS1_targeting_F and IWS1_targeting_R, SUB2_targeting_F and SUB2_targeting_R, RAMP4_targeting_F and RAMP4_targeting_R, and DRL1_targeting_F and DRL1_targeting_R.

10.1128/mbio.03256-22.8 TABLE S3 List of primers used in this study. Primer names, restriction enzymes (if available), sequences, and resulting plasmids/descriptions are shown. Download Table S3, PDF file, 0.3 MB. Copyright © 2023 Hashizaki et al. 2023 Hashizaki et al. https://creativecommons.org/licenses/by/4.0/ This content is distributed under the terms of the Creative Commons Attribution 4.0 International license.

### Generation of gene-targeted *T. gondii* strains by CRISPR/Cas9 genome editing.

RHΔ*hxgprt*Δ*ku80* strain parasites expressing firefly luciferase were filtered, washed, and resuspended in Cytomix (10 mM KPO_4_, 120 mM KCl, 0.15 mM CaCl_2_, 5 mM MgCl_2_, 25 mM HEPES, 2 mM EDTA). Parasites were mixed with 50 μg of each gRNA1 and gRNA2 CRISPR plasmid for each gene, along with the PCR-amplified targeting fragment for each gene, and supplemented with 2 mM ATP and 5 mM glutathione (GSH). Parasites were electroporated by Gene Pulser II (Bio-Rad Laboratories). Selection by growth for 14 days in 25 μg/mL mycophenolic acid (Sigma) and 50 μg/mL xanthine (Wako) were used to obtain stably resistant clones. Next, parasites were plated in limiting dilution in 96-well plates to isolate single clones. To confirm the disruption of the gene, we analyzed mRNA of IWS1, SUB2, RAMP4 or DRL1 from WT and each KO (knockout) parasite by quantitative RT-PCR using the primers listed in Table S3.

### Complementation of IWS1 in IWS1-deficient *T. gondii*.

To remove the HXGPRT gene expression cassette, parasites were transiently transfected with Cre expression vectors. Selection by growth for 14 days in 80 μg/mL 6-thioxanthine (Wako) was used to obtain stably resistant clones. A total of 24 clones were selected and screened by PCR to detect deletion of the HXGPRT gene expression cassette. To complement the IWS1-deficient parasites, the IWS1 coding region was amplified from RH T. gondii genomic DNA using the primers IWS1_cDNA_F and IWS1_cDNA_R (Table S3), subcloned into pCR-Blunt II TOPO (Thermo Fisher Scientific), and sequenced. The plasmid was cut by BglII and PacI and the IWS1 cDNA fragment was inserted into the BamHI/PacI site of *hxgprt* cassette containing poly A signal at the 5′ side of the cassette. To construct CRISPR/Cas9 plasmids for complementing IWS1, two oligonucleotide primers (IWS1_complement_gRNA3_F and IWS1_complement_gRNA3_R) were used. To insert the IWS1-polyA-HXGPRT gene cassette into the endogenous locus of IWS1 (Fig. S2A), the cassette was amplified using the primers IWS1_complement_F and IWS1_complement_R. IWS1-deficient parasites lacking HXGPRT were filtered, washed and resuspended in Cytomix. Parasites were mixed with 50 μg of the IWS1-complement_gRNA3 along with the PCR-amplified targeting fragment and supplemented with 2 mM ATP and 5 mM GSH. Parasites were electroporated using a Gene Pulser II. Selection by growth for 14 days in 25 μg/mL mycophenolic acid (Sigma) and 50 μg/mL xanthine (Wako) was used to obtain stably resistant clones. Next, parasites were plated in limiting dilution in 96-well plates to isolate single clones. To confirm expression of the gene encoding IWS1, we analyzed IWS1 mRNA from the rescued parasites by quantitative RT-PCR using the primers listed in Table S3.

### Overexpression of ROP18 in IWS1-deficient *T. gondii*.

The IWS1-deficient parasites lacking HXGPRT were filtered, washed, and resuspended in Cytomix. Parasites were mixed with 50 μg of the ROP18 expression vector containing ROP18 cDNA (18), in which 1 kb promoter of the *SAG1* gene was fused to the coding sequence of ROP18 cDNA, followed by the poly A sequence and *hxgprt* expression cassette in pBlueScript plasmid vector (Fig. S2B), and supplemented with 2 mM ATP and 5 mM GSH. Parasites were electroporated using a Gene Pulser II. Selection by growth for 14 days in 25 μg/mL mycophenolic acid (Sigma) and 50 μg/mL xanthine (Wako) was used to obtain stably resistant clones. Then, parasites were plated in limiting dilution in 96-well plates to isolate single clones. To confirm expression of *rop18*, we analyzed ROP18 mRNA from the rescued parasites by quantitative RT-PCR using the primers listed in Table S3.

### Immunofluorescence assay.

MEFs were plated at equal densities (1.5 × 10^5^ cells per well in a 6-well plate) on glass coverslips and exposed to 10 ng/mL IFN-γ for 18 to 20 h at 37°C. Cells were infected with T. gondii at MOI = 4 and incubated at 37°C for 2 h (Fig. 1, 2, and 5 and Fig. S3) and 48 h (Fig. S5). Next, cells were fixed in phosphate-buffered saline (PBS) containing 3.7% paraformaldehyde for 10 min. Cells were then permeabilized with 0.002% digitonin (Fig. 1, 2, and 5 and Fig. S3) or 0.1% Triton (Fig. S5) in PBS for 10 min and blocked with 8% FBS in PBS for 60 min.

Cells were co-stained with anti-GRA7 or anti-GRA2 and antibodies against Irgb6, Irga6, GBP2, p62 or ubiquitin for 1 h, followed by incubation with Alexa 488- and Alexa 594-conjugated secondary antibodies and DAPI (4′,6-diamidino-2-phenylindole) for 30 min in the dark. Coverslips were mounted onto glass slides with PermaFluor and analyzed with confocal laser microscopy (Olympus FV3000 IX83). All procedures following the 2-h incubation were done at room temperature. All images were taken with a 60× lens objective.

### Assessment of *in vivo* virulence in mice.

Mice were intraperitoneally infected with 1.0 × 10^3^ T. gondii tachyzoites in 200 μL PBS per mouse. Survival rates were measured daily for 25 days.

### *In vivo* measurement of parasites by bioluminescent imaging.

Mice were intraperitoneally infected with 1.0 × 10^3^ T. gondii tachyzoites in 200 μL PBS per mouse. *In vivo* imaging was done on day 6 postinfection. Mice were intraperitoneally injected with 3 mg d-luciferin (Promega) in 200 μL PBS, anesthetized for 10 min with isoflurane (Dainippon Sumitomo Pharma), and used for photonic emission detection with IVIS-Spectrum and Living Image Software (Xenogen).

### Organ luciferase assay.

Mice were dissected on day 6 postinfection. Peritoneal exudate cells (PEC) were collected with PBS. Organs were homogenized in 1× Promega Passive Lysis Buffer. Next, 200 μL of PEC and organ homogenates were collected, sonicated for 30 s, and centrifuged for 10 min at 13,000 rpm at 4°C. A 10-μL volume of each supernatant was mixed with 50 μL LARII (Promega Dual-Luciferase Reporter Assay System) and used for luciferase activity detection with a Promega GLOMAX20/20 luminometer.

### Plaque assay.

Two hundred tachyzoites were added onto a 6-well plate covered with primary MEFs and incubated at 37°C in 5% CO_2_ for 7 days. The cells were washed with PBS, fixed for 30 min with paraformaldehyde, then stained with 1% crystal violet. Plaque numbers were counted after full washing with water. Photos were taken at 4× magnification with an optical microscope (Olympus CKX53).

### *In vitro* measurement of *T. gondii* numbers by luciferase assay.

MEFs were seeded at 1.0 × 10^5^ cells per well in a 12-well plate, treated or untreated with 10 ng/mL IFN-γ, and incubated at 37°C for 18 to 20 h. Luc-expressing tachyzoites were added to these cells (MOI = 1) and incubated at 37°C for 22 to 24 h. Cells were collected and centrifuged (4°C at 7,000 rpm for 5 min) and suspended in 100 μL 1× Promega Passive Lysis Buffer, followed by sonication for 30 s. Cells were then centrifuged (4°C at 14,000 rpm for 5 min), and 5 μL of supernatants was mixed with 50 μL LARII and used for luciferase activity detection with a Promega GLOMAX20/20 luminometer. Data shown represent luciferase activity relative to that in untreated MEFs.

### Gene expression measurement by quantitative PCR.

Tachyzoites were collected and centrifuged (room temperature, 2,000 rpm, 5 min), followed by total RNA extraction with a Qiagen RNeasy Minikit. RNA samples were incubated at 70°C for 2 to 3 min and used to synthesize cDNA with a Verso cDNA Synthesis kit (Thermo Fisher Scientific). RT-PCR was performed with the synthesized cDNA and Promega GoTaq qPCR Master Mix using the Bio-Rad CFX Connect Real-Time System. All expression data were taken using the 2^ΔΔ^*^CT^* (threshold cycle) method relative to TgACT1 (TGGT1_209030) expression levels.

### RNA-seq analysis.

For RNA-seq analysis, total RNA was extracted from cells using a miRNeasy minikit (Qiagen) following the manufacturer’s instructions. Library preparation was performed using a TruSeq stranded mRNA sample prep kit (Illumina, San Diego, CA) according to the manufacturer’s instructions. Sequencing was performed on an Illumina NovaSeq 6000 sequencer (Illumina) in 101-base single-end mode. Sequenced reads were mapped to the T. gondii GT1 reference genome sequences (GCA_000149715.2_TGGT1) using HISAT2 version 2.1.0 (31), and reads aligned to the *Toxoplasma* reference genome were counted using featureCounts (32). Raw count data were analyzed using iDEP.92 (33) and GSEA software version 4.1.0. (34). Genes with a false discovery rate (FDR) of 0.05 and |log_2_-fold change| > 1 were considered differentially expressed. For GSEA, gene sets were obtained from published data. Heatmaps visualizing normalized gene expression levels on a scale of rawmin to rawmax for each gene were generated using Morpheus (https://software.broadinstitute.org/morpheus).

### ChIP-seq analysis.

To generate the N-terminal, Venus-expressing IWS1 knock-in (N-Venus-IWS1-KI) parasites, the *hxgprt* cassette followed by the 1-kb SAG1 promoter and Venus without stop codon was amplified by PCR with the following primers which contain 60-bp homology arms of the IWS1 locus (IWS1_NVenus_KI_F and IWS1_NVenus_KI_R) (Fig. S5A and Table S3). WT parasites lacking HXGPRT were filtered, washed, and resuspended in Cytomix. Parasites were mixed with 50 μg of the IWS1-complement_gRNA3 along with the PCR-amplified targeting fragment and supplemented with 2 mM ATP and 5 mM GSH. Parasites were electroporated using a Gene Pulser II. Selection by growth for 14 days in 25 μg/mL mycophenolic acid (Sigma) and 50 μg/mL xanthine (Wako) was used to obtain stably resistant clones. N-terminal Venus-tagged IWS1 expression was confirmed by Western blotting (data not shown) and immunofluorescence assay (IFA) in the N-Venus-IWS1-KI parasites, but not in the control parasites expressing non-tagged Venus alone (Fig. S5B). ChIP was performed with a Chromatin Immunoprecipitation Assay kit (Millipore) according to the manufacturer’s protocol. The obtained chromatin was sonicated with a Bioruptor (Cosmo Bio Co., Ltd.) in a lysis solution at 4°C for 12.5 min (H-amplitude; 10-s sonication at 20-s intervals). Chromatin was immunoprecipitated with polyclonal anti-GFP rabbit antibody which also recognizes Venus (Abcam). Input DNAs from N-Venus-IWS1-KI parasites or negative-control Venus alone-expressing parasites were obtained from chromatin without IP. After immunoprecipitation with the anti-GFP antibody, DNA was detected in the samples from N-Venus-IWS1-KI parasites but not in those from Venus alone-expressing parasites (data not shown). Therefore, the input and immunoprecipitated DNAs from N-Venus-IWS1-KI parasites were sequenced with a NovaSeq 6000 sequencer (Illumina). Read sequences were mapped onto the reference T. gondii GT-1 genome under stringent conditions, allowing no mismatches within 60 bp, using bowtie2 (version 2.3.5.1). The data have been deposited in the DNA Data Bank of Japan (DDBJ) under accession no. DRA015179. The mapping data (IP and input) were analyzed with the MACS2 peak-calling algorithm (FDR = 0.01 and fold enrichment > 2), using ~5.4 × 10^8^ reads for IP and 3.5 × 10^8^ reads for the input control in experiment 1, ~5.0 × 10^8^ for IP and 3.4 × 10^7^ for input control in experiment 2, and ~5.2 × 10^8^ for IP and 4.6 × 10^7^ for input control in experiment 3. Peaks in experiment 1 which showed a counterpart within 100 bp of a peak in experiments 2 and 3 were selected as common peaks that were common to the three replicates. Target genes were predicted from the common peaks using in-house software.

### Statistical analysis.

Three points in all graphs represent three means derived from three independent experiments (three biological replicates). All statistical analyses were performed using Prism 9 (GraphPad). In infection assays, differences in T. gondii inhibition activity between IFN-γ versus Medium were subjected to two-way analysis of variance (ANOVA) with Tukey’s multiple-comparison test to analyze differences between genotypes (Fig. 5D). When comparing percentages of Irgb6 recruitment and expression levels of ROP18 or IWS1 between different genotypes, ordinary one-way ANOVA was used (Fig. 1B, Fig. 4D, and Fig. 5A). The statistical significance of differences in mouse survival between two groups was assessed using a Kaplan-Meier survival analysis and a log-rank test (Fig. 3C and E, Fig. 6B).
