# Supplementary figures and images for: Toxoplasma IWS1 Determines Fitness in Interferon-γ-Activated Host Cells and Mice by Indirectly Regulating ROP18 mRNA Expression
Source: mBio. 2023 Jan 30;14(1):e03256-22. doi: 10.1128/mbio.03256-22 (PMC9973038; doi:10.1128/mbio.03256-22)

# Supplementary Figure 1\_Hashizaki et al.

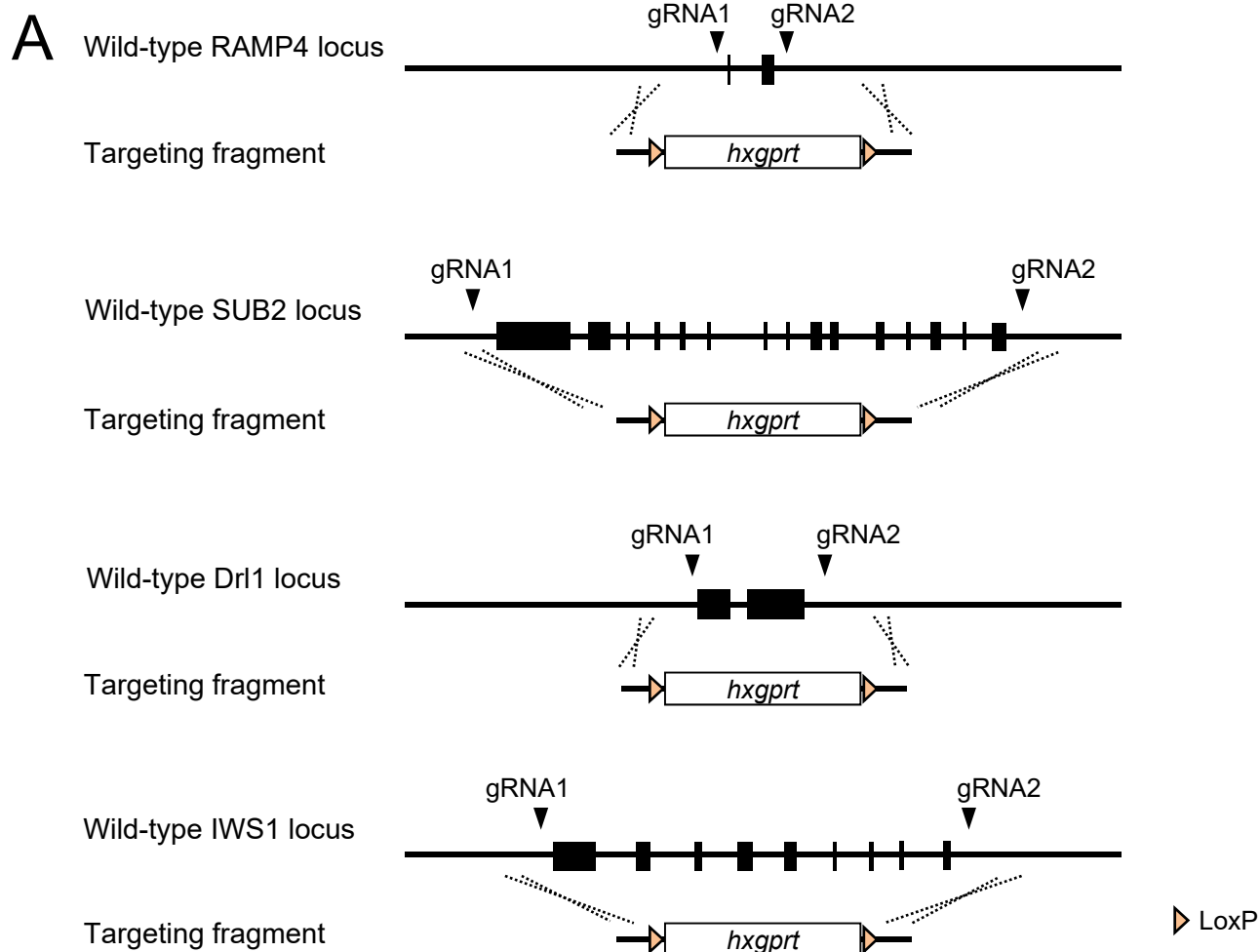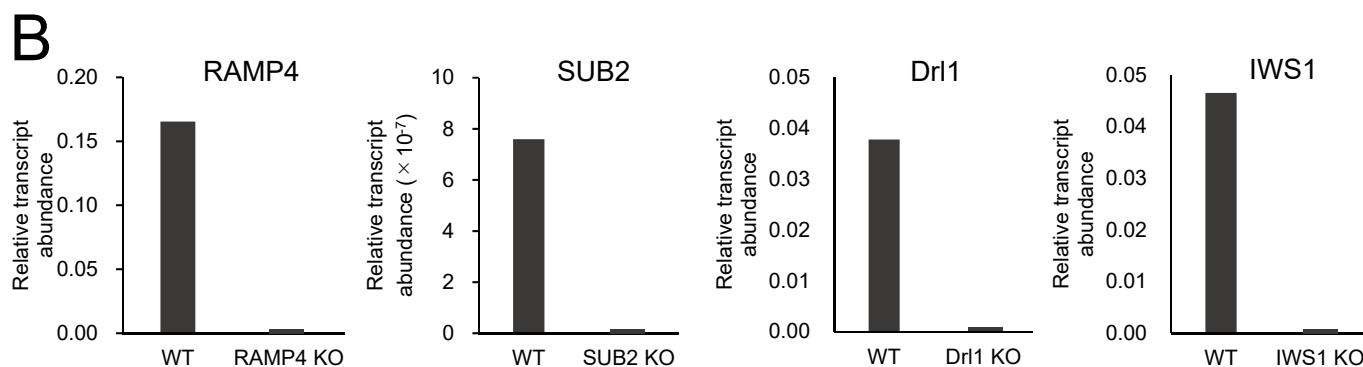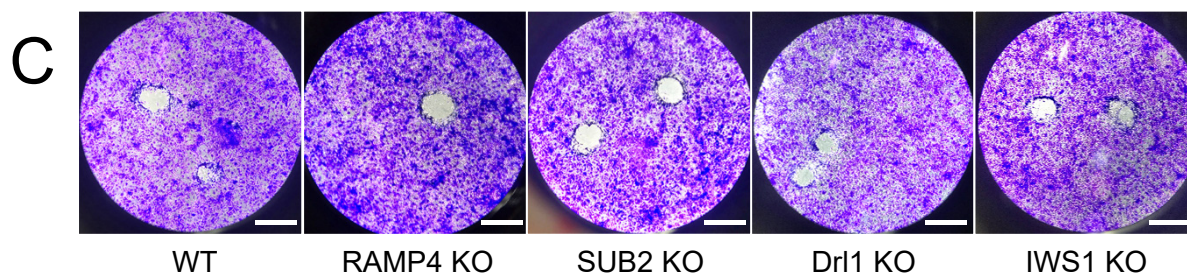

Supplement: FIG S1 [file mbio.03256-22-s0001.pdf]

# Supplementary Figure 2\_Hashizaki et al.

A

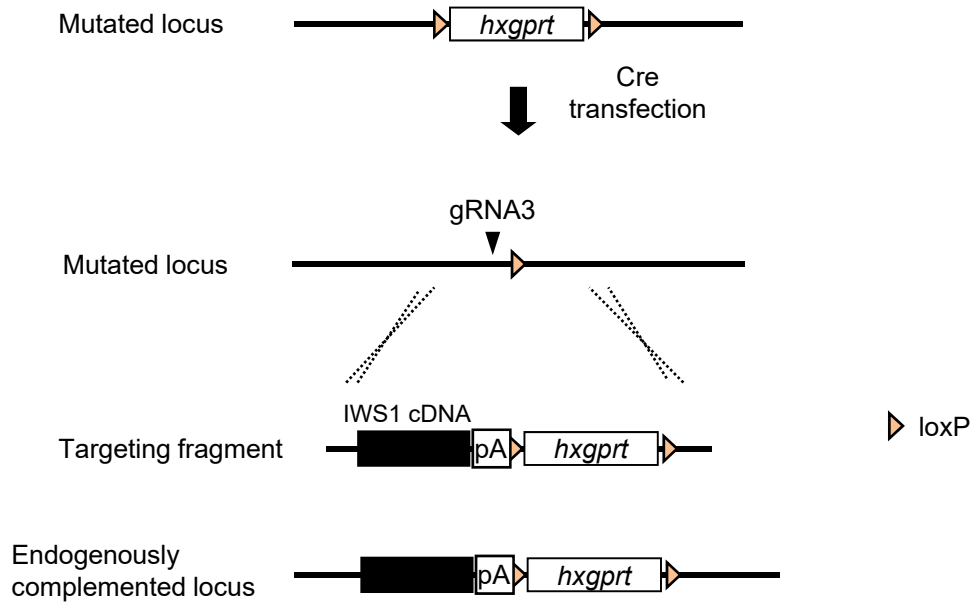

B

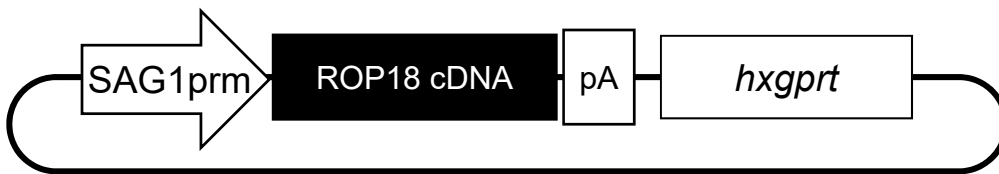

Supplement: FIG S2 [file mbio.03256-22-s0002.pdf]

# Supplementary Figure 3\_Hashizaki et al.

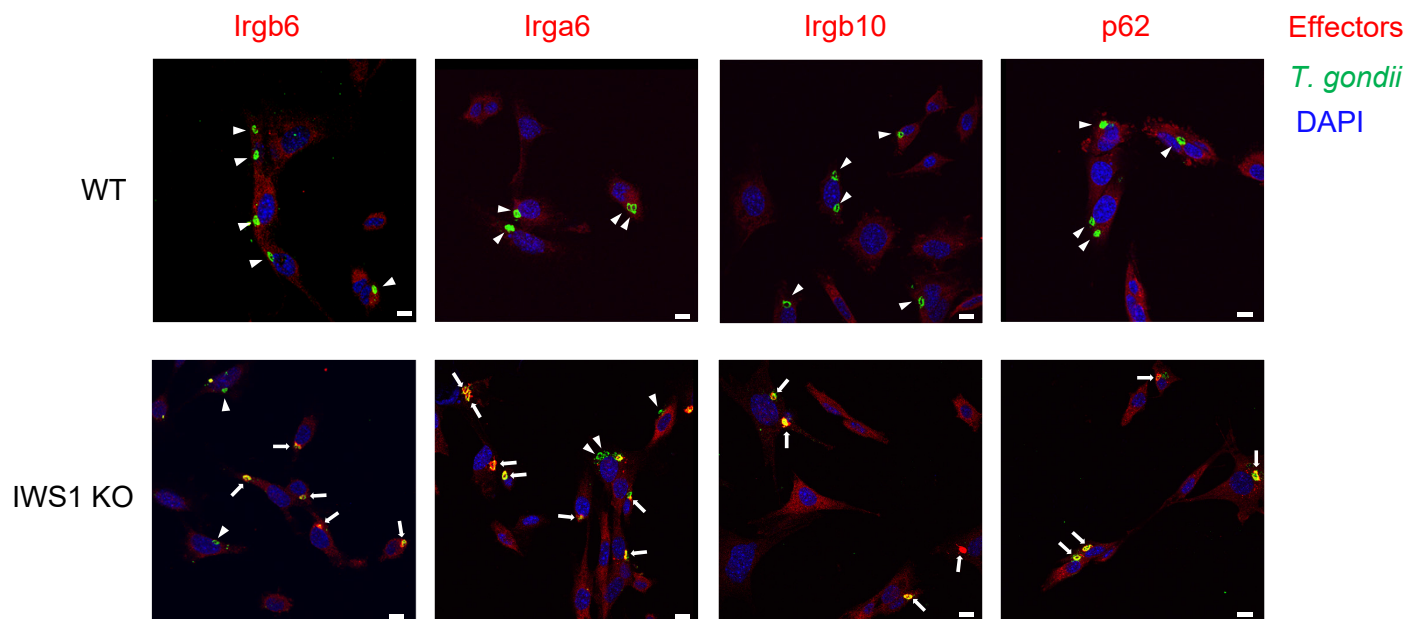

Supplement: FIG S3 [file mbio.03256-22-s0003.pdf]

# Supplementary Figure 5\_Hashizaki et al.

A

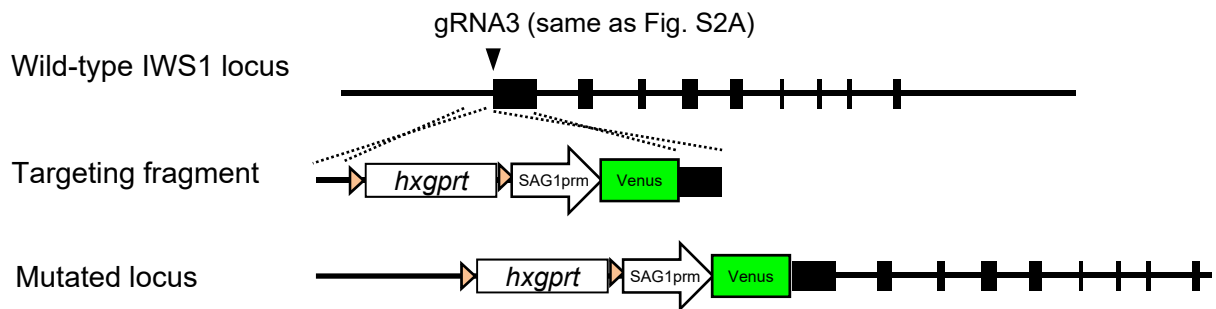

B

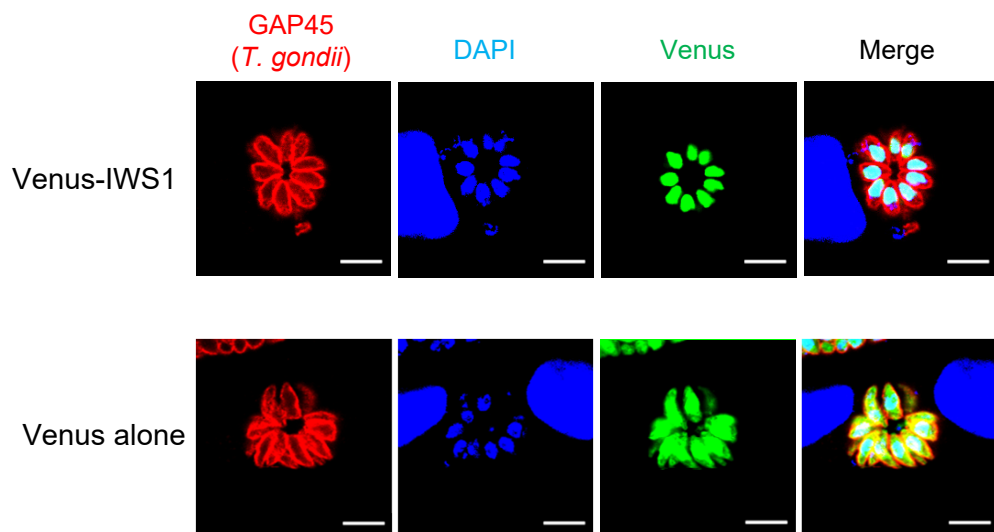

C

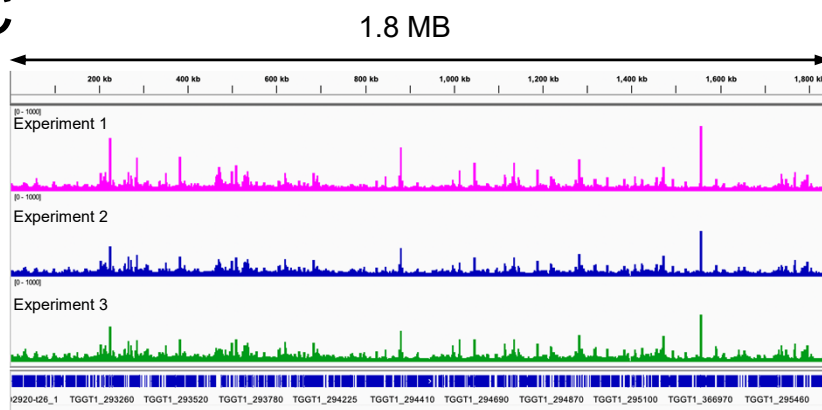

D

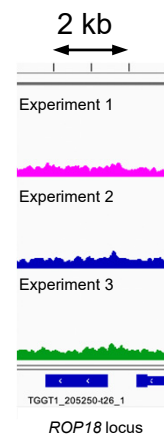

Supplement: FIG S5 [file mbio.03256-22-s0005.pdf]
