# Supplementary material for: Toxoplasma IWS1 Determines Fitness in Interferon-γ-Activated Host Cells and Mice by Indirectly Regulating ROP18 mRNA Expression
Source: mBio. 2023 Jan 30;14(1):e03256-22. doi: 10.1128/mbio.03256-22 (PMC9973038; doi:10.1128/mbio.03256-22)
Supplement: FIG S4 [file mbio.03256-22-s0004.pdf]

Supplementary Figure 4_Hashizaki et al.

Fold change	Gene ID	Protein
1162.242	TGGT1_227560	putative IWS1 transcription factor
135.788	TGGT1_320740	hypothetical protein
93.351	TGGT1_221310	aminopeptidase N protein
29.255	TGGT1_253300	hypothetical protein
21.302	TGGT1_306334	hypothetical protein
19.978	TGGT1_360210	toxoplasma gondii family B protein
16.213	TGGT1_238165	hypothetical protein
15.939	TGGT1_297643	hypothetical protein
14.259	TGGT1_216290B	hypothetical protein
14.159	TGGT1_285870	SAG-related sequence SRS20A
13.637	TGGT1_278930	SAG-related sequence SRS20A
12.166	TGGT1_254350	endo-1,3(4)-beta-glucanase
10.760	TGGT1_317705	enoyl-CoA hydratase/isomerase family protein
10.042	TGGT1_286150	PAN/Apple domain-containing protein
9.736	TGGT1_411840	hypothetical protein
9.316	TGGT1_205250	rhoptry protein ROP18
8.438	TGGT1_301890	Toxoplasma gondii family B protein
8.131	TGGT1_307030	Purine nucleoside phosphorylase
7.988	TGGT1_203720	vitamin k epoxide reductase family protein
7.463	TGGT1_254360	hypothetical protein
7.198	TGGT1_311460	hypothetical protein
6.221	TGGT1_297647	hypothetical protein
6.184	TGGT1_285470	patched family protein
6.095	TGGT1_229140	MaoC family domain-containing protein
5.635	TGGT1_216335	hypothetical protein
5.583	TGGT1_306338B	putative dynein gamma chain, flagellar outer arm
5.320	TGGT1_213430	hypothetical protein
5.018	TGGT1_277800	hypothetical protein
4.824	TGGT1_239090	SAG-related sequence SRS23
4.807	TGGT1_254855	hypothetical protein
4.736	TGGT1_296020	hypothetical protein
4.732	TGGT1_312930	putative cystathione gamma lyase
4.642	TGGT1_204050	subtilisin SUB1
4.574	TGGT1_278120	SCP family extracellular subfamily protein
4.550	TGGT1_225950	hypothetical protein
