# Supplementary material for: Toxoplasma IWS1 Determines Fitness in Interferon-γ-Activated Host Cells and Mice by Indirectly Regulating ROP18 mRNA Expression
Source: mBio. 2023 Jan 30;14(1):e03256-22. doi: 10.1128/mbio.03256-22 (PMC9973038; doi:10.1128/mbio.03256-22)
Supplement: TABLE S3 [file mbio.03256-22-s0008.pdf]

Table S3_Hashizaki et al.

Table S3. Primers used in this study.

Primer name	Enzyme	Sequence	Resulting plasmids and descriptions
IWS1_gRNA1_F	—	5’-AAGTTgtatggcggacgcgggagacgG -3’	IWS1 gRNA1
IWS1_gRNA1_R	—	5’-AAAACcgtctcccgcgtccgccatacA -3’	IWS1 gRNA1
IWS1_gRNA2_F	—	5’- AAGTTggcgaagtcttcaccgtttcaG -3	IWS1 gRNA2
IWS1_gRNA2_R	—	5’-AAAACtgaaacggtgaagacttcgccA -3’	IWS1 gRNA2
SUB2_gRNA1_F	—	5’- AAGTTgctcatttaggtcaccgaagaG-3’	SUB2 gRNA1
SUB2_gRNA1_R	—	5’- AAAACtcttcggtgacctaaatgagcA -3’	SUB2 gRNA1
SUB2_gRNA2_F	—	5’- AAGTTgacgagaatgagtcgtagcctG -3’	SUB2 gRNA2
SUB2_gRNA2_R	—	5’- AAAACaggctacgactcattctcgtcA -3’	SUB2 gRNA2
DRL1_gRNA1_F	—	5’-AAGTTggtgtgagtctcggtcccgcgG-3’	DRL1 gRNA1
DRL1_gRNA1_R	—	5’-AAAACcgcgggaccgagactcacaccA-3’	DRL1 gRNA1
DRL1_gRNA2_F	—	5’-AAGTTgcgtcgctggacctgcccgaaG-3’	DRL1 gRNA2
DRL1_gRNA2_R	—	5’-AAAACttcgggcaggtccagcgacgcA-3’	DRL1 gRNA2
RAMP4_gRNA1_F	—	5’-AAGTTggtctgcttggcttccagcaaG-3’	RAMP4 gRNA1
RAMP4_gRNA1_R	—	5’-AAAACttgctggaagccaagcagaccA-3’	RAMP4 gRNA1
RAMP4_gRNA2_F	—	5’-AAGTTgggagcgttcaactattatgtG-3’	RAMP4 gRNA2
RAMP4_gRNA2_R	—	5’-AAAACacataatagttgaacgctcccA-3’	RAMP4 gRNA2
IWS1_targeting_F	—	5’-ccggaatctcttgccctaagcgttcgtgacgcgtctcgagtatggcggacgcgggagacgATAACTTCGTATAGCATACATTATACGAAGTTATagcttgatcagcacgaaaccttgca- 3’	Generation of IWS1 KO parasites
IWS1_targeting_R	—	5’-cacgtccttcaattgaaaccttctgcgctctggtcggcttccgaactgatcgtgcatccaATAACTTCGTATAATGTATGCTATACGAAGTTATgatccccctccaccgcggtgtcact-3’	Generation of IWS1 KO parasites
SUB2_targeting_F	—	5’-tgtgagacgtactgggcagctatgcatcttccccaccttgctcatttaggtcaccgaagaATAACTTCGTATAGCATACATTATACGAAGTTATagcttgatcagcacgaaaccttgca-3’	Generation of SUB2 KO parasites
SUB2_targeting_R	—	5’-aatatccaacacgccttcaaggacgttgccgatagactgcgcatcacacgccttctaccaATAACTTCGTATAATGTATGCTATACGAAGTTATgatccccctccaccgcggtgtcact-3’	Generation of SUB2 KO parasites
DRL1_targeting_F	—	5’-acatgagcgcatgcagcgattcgtcctgcgaaaatttctcgtgtgagtctcggtcccgcgATAACTTCGTATAGCATACATTATACGAAGTTATagcttgatcagcacgaaaccttgca-3’	Generation of DRL1 KO parasites
DRL1_targeting_R	—	5’-gggagggaagagtgctcgaacggttaacgtagaaattcagtctcgcggggagtgcatccgATAACTTCGTATAATGTATGCTATACGAAGTTATgatccccctccaccgcggtgtcact-3’	Generation of DRL1 KO parasites
RAMP4_targeting_F	—	5’-agtgactcttcttttttcctccagctttagcaaggattgtgtctgcttggcttccagcaaATAACTTCGTATAGCATACATTATACGAAGTTATagcttgatcagcacgaaaccttgca-3’	Generation of RAMP4 KO parasites
RAMP4_targeting_R	—	5’-tctgaagtcgaggatcaacttctgctgctactgatccttatttctgcacgtgattctccaATAACTTCGTATAATGTATGCTATACGAAGTTATgatccccctccaccgcggtgtcact-3’	Generation of RAMP4 KO parasites
IWS1_complement_gRNA3_F	—	5’-AAGTTggtgacgcgtctcgagtatggG-3’	gRNA3 for IWS1_complementation
IWS1_complement_gRNA3_R	—	5’-AAAACccatactcgagacgcgtcaccA-3’	gRNA3 for IWS1_complementation
IWS1_cDNA_F	BglII	AGATCTACCATGGCGGACGCGGGAGACGAGGGAGATGCCCTCAGGGCAAA-3’	IWS1 cDNA
IWS1_cDNA_R	PacI	5’-ttaattaaCTActtgtcgtcatcgtctttgtagtcGAGGCCACGTCCTTCAATTGAAACCTTCTG	IWS1 cDNA
IWS1_complement_F	—	5’-CTCGGACCTCGTTTTTTTTCCGGAATCTCTTGCCCTAAGCGTTCGTGACGCGTCTCGAGTaccATGGCGGACGCGGGAGACGAGGGAGATGCCCTCAGGGCAAATGCAGGAGAGTCACCC- 3’	IWS1 complementation in IWS1 KO parasites
IWS1_complement_F	—	5’-cacgtccttcaattgaaaccttctgcgctctggtcggcttccgaactgatcgtgcatccaTCTAGAACTAGTGGATCAGATCCCCCTCGGGGGGGCAAGAATTGTGTTAACCGGTTCGAC- 3’	IWS1 complementation in IWS1 KO parasites
TgIWS1_qPCR_F	—	5’-AATGCGGCAAAGGAAACGAC-3’	Quantitative RT-PCR
TgIWS1_qPCR_R	—	5’-GAGTGGAGGCATCGGACATT-3’	Quantitative RT-PCR
TgSUB2_qPCR_F	—	5’-TGGAGCACGCATCAAAGGAT-3’	Quantitative RT-PCR
TgSUB2_qPCR_R	—	5’-CCAAAGCTCTGGGTTTTGCC-3’	Quantitative RT-PCR
TgDRL1_qPCR_F	—	5’-AAAATGTGTGGTGGTCCCGA-3’	Quantitative RT-PCR
TgDRL1_qPCR_R	—	5’-TCGGTTTCTGACAAGGACGG-3’	Quantitative RT-PCR
TgRAMP4_qPCR_F	—	5’-GTGGCAATGTTCCGGAAAGC-3’	Quantitative RT-PCR
TgRAMP4_qPCR_R	—	5’-AATCGCAGATCCCACAACGA-3’	Quantitative RT-PCR
TgROP18_qPCR_F	—	5’-ACTGCATCTGGTGCAAGGAA-3’	Quantitative RT-PCR
TgROP18_qPCR_R	—	5’-AGAGGCTTCGGATGAGGTCT-3’	Quantitative RT-PCR
TgACT1_qPCR_F	—	5’-TGGATCGGAGGGTCCATTCT-3’	Quantitative RT-PCR
TgACT1_qPCR_R	—	5’-TCGTCGTACTCCTCCTTGGT-3’	Quantitative RT-PCR
IWS1_NVenus_KI_F	—	5’-GGACCTCGTTTTTTTTCCGGAATCTCTTGCCCTAAGCGTTCGTGACGCGTCTCGAGTATGtgatcagcacgaaaccttgcattcaaacccgcccgcggaagatccgatcttgctgctgtt - 3’	Generation of the N-ternimal-Venus-IWS1 KI parasites
IWS1_NVenus_KI_R	—	5’-tggtctgggtgactctcctgcatttgccctgagggcatctccctcgtctcccgcgtccgcCAGCTCGTCCATGCCGAGAGTGATCCCGGCGGCGGTCACGAACTCCAGCAGGACCATGTG - 3’	Generation of the N-ternimal-Venus-IWS1 KI parasites
